# Comparative transcriptome analysis and RNA interference reveal *CYP6A8* and SNPs related to pyrethroid resistance in *Aedes albopictus*

**DOI:** 10.1371/journal.pntd.0006828

**Published:** 2018-11-12

**Authors:** Jiabao Xu, Xinghua Su, Mariangela Bonizzoni, Daibin Zhong, Yiji Li, Guofa Zhou, Hoan Nguyen, Sarah Tong, Guiyun Yan, Xiao-Guang Chen

**Affiliations:** 1 Key Laboratory of Prevention and Control for Emerging Infectious Diseases of Guangdong Higher Institutes, Department of Pathogen Biology, School of Public Health, Southern Medical University, Guangzhou, China; 2 Department of Biology and Biotechnology, University of Pavia, Pavia, Italy; 3 Program in Public Health, University of California, Irvine, Irvine, CA, United States of America; 4 Key Laboratory of Translational Medicine Tropical Diseases of Ministry of Education and Department of Pathogen Biology, Hainan Medical University, Haikou, Hainan, China; University of California Davis, UNITED STATES

## Abstract

Wide and improper application of pyrethroid insecticides for mosquito control has resulted in widespread resistance in *Aedes albopictus* mosquitoes, an important dengue vector. Therefore, understanding the molecular regulation of insecticide resistance is urgently needed to provide a basis for developing novel resistance diagnostic methods and vector control approaches. We investigated the transcriptional profiles of deltamethrin-resistant and -susceptible *Ae*. *albopictus* by performing paired-end sequencing for RNA expression analysis. The analysis used 24 independent libraries constructed from 12 wild-caught resistant and 12 susceptible *Ae*. *albopictus* female adults. A total of 674,503,592 and 612,512,034 reads were obtained, mapped to the *Ae*. *albopictus* genome and assembled into 20,091 *Ae*. *albopictus* transcripts. A total of 1,130 significantly differentially expressed genes included 874 up-regulated genes and 256 down-regulated genes in the deltamethrin-resistant individuals. These differentially expressed genes code for cytochrome P450s, cuticle proteins, glutathione S-transferase, serine proteases, heat shock proteins, esterase, and others. We selected three highly differentially expressed candidate genes, *CYP6A8* and two genes of unknown function (CCG013931 and CCG000656), to test the association between these 3 genes and deltamethrin resistance using RNAi through microinjection in adult mosquitoes and oral feeding in larval mosquitoes. We found that expression knockdown of these three genes caused significant changes in resistance. Further, we detected 1,162 single nucleotide polymorphisms (SNPs) with a frequency difference of more than 50%. Among them, 5 SNPs in 4 cytochrome P450 gene families were found to be significantly associated with resistance in a genotype-phenotype association study using independent field-collected mosquitoes of known resistance phenotypes. Altogether, a combination of novel individually based transcriptome profiling, RNAi, and genetic association study identified both differentially expressed genes and SNPs associated with pyrethroid resistance in *Ae*. *albopictus* mosquitoes, and laid a useful foundation for further studies on insecticide resistance mechanisms.

## Author summary

*Aedes albopictus* represents a serious threat to public health as a vector for a number of arboviruses, such as dengue, Zika, and chikungunya viruses. Its ability to resist insecticides has recently been detected in several countries. However, little is known regarding molecular mechanisms that confer pyrethroid resistance. Determining such mechanisms is essential for early detection, monitoring, and management of insecticide resistance. Here, we use high-throughput RNA sequencing techniques, RNAi, and genotype-phenotype association study to characterize qualitative and quantitative differences in gene expression as well as single nucleotide polymorphisms between resistant and susceptible *Ae*. *albopictus* individuals collected from a dengue-endemic area in southern China. We identified 3 candidate genes and 5 candidate SNPs associated with deltamethrin resistance. These results enhance the understanding of insecticide resistance mechanisms among *Aedes albopictus*.

## Introduction

Arboviruses such as Zika, dengue, and Chikungunya by *Aedes* mosquitoes represent an expanding threat to global health [1–3]. Chemical control through the use of insecticides is one of the essential tools in the global strategy for mosquito-borne infectious disease control [4]. Pyrethroid is often the insecticide of choice due to its low mammalian toxicity and high insecticidal activity [4,5]. Permethrin, deltamethrin, cypermethrin, and cyfluthrin are currently the most commonly used pyrethroids for adult mosquito control in indoor residual spraying (IRS), insecticide-treated nets (ITN), and space spray treatment [6, 7]. For instance, ultra-low-volume (ULV) spray of pyrethroids has been a major measure for controlling *Aedes* adults in dengue-endemic areas in China [8]. However, excessive and improper application of pyrethroids for mosquito control, together with widespread agricultural usage, has resulted in a high selection pressure for pyrethroid resistance [9,10]. Insecticide resistance has become a major impediment to controlling mosquito-borne diseases worldwide. As a response to this dilemma, the World Health Organization (WHO) launched the Global Plan for Insecticide Resistance Management (GPIRM) to guide rational insecticide usage [11]. Insecticide resistance in the Asian tiger mosquito, *Aedes albopictus*, the major dengue vector, came to public attention in China with the dengue outbreak in 2014, when over 45,000 dengue cases were reported in Guangdong province [12]. Insecticide was applied extensively under the government-directed mosquito control program, and resistance has been reported in multiple *Ae*. *albopictus* populations in Guangdong [13]. Current resistance monitoring relies on bioassays with live mosquitoes. Understanding resistance mechanisms is essential to developing sensitive molecular monitoring tools of resistance and resistance management.

Resistance to DDT and pyrethroids has been documented in different mosquito species [10,14,15]. Two major resistance mechanisms have been previously identified, including target site insensitivity resulting from mutations in the insecticides’ target protein genes and increased metabolic detoxifications for insecticides [4]. Non-synonymous mutations in the voltage-gated sodium channel (VGSC) gene causing target-site insensitivity have been found in multiple mosquito species of public health importance, including *Anopheles gambiae* [16,17], *Culex quinquefasciatus* [15,18], *Ae*. *aegypti* [10,14], and *Ae*. *albopictus* [13]. In *Ae*. *aegypti*, V1016G, V1016I, and F1534C mutations were found to be correlated with pyrethroid resistance [19, 20], and the F1534S mutation was correlated with deltamethrin resistance in *Ae albopictus* [13]. Metabolic resistance involves the insecticide molecule’s bio-transformation via higher expression or presence of more efficient detoxification enzymes. Detoxification enzymes belong to four gene families: cytochrome P450 monooxygenases (P450s), carboxylesterases (COEs), glutathione S-transferases (GSTs), and UDP-glucuronosyltransferases, but a number of species-specific genes within each gene family have been found to be involved in metabolic resistance in various mosquito species, such as *Anopheles sinensis*, *Cx*. *quinquefasciatus*, and *Ae*. *aegypti* [10,14,15,17,21–24]. Other resistance mechanisms include mosquito physiological changes, such as cuticle thickening and digestive tract modification, which may lead to changes in insecticide absorption and penetration, and in behavioral avoidance [25, 26]. We have previously identified one mutation (F1534S) in the VGSC gene in *Ae*. *albopictus* that is significantly associated with pyrethroid resistance [13]. However, little is known about metabolic resistance in *Ae*. *albopictus*, which may result from both altered expression and mutations in the detoxification genes. For example, SNPs in *GSTe*2 were found to confer resistance to DDT in the malaria mosquito *Anopheles funestus* [27]. With this in mind, this study aims to characterize novel resistance mechanisms in *Ae*. *albopictus*.

mRNA sequencing (RNA-seq) is a powerful technique for comprehensive profiling of transcripts. To date, several studies have used RNA-seq to investigate resistance mechanisms in mosquito species, including *An*. *gambiae* [28], *An*. *sinensis* [29], *Ae*. *aegypti* [30], and *Cx*. *quinquefasciatus* [31] and *Cx*. *pipiens pallens* [32], and these studies have demonstrated that the RNA-seq technique can successfully yield useful resistance targets. In southern China, *Ae*. *albopictus* is the most important dengue vector with strong pyrethroid resistance [13]. In this context, the present study used RNA-seq on single mosquitoes and identified genes differentially expressed between resistant and susceptible *Ae*. *albopictus* mosquitoes and SNPs associated with resistance. In addition, the function of 3 candidate genes was further functionally verified by RNA interference (RNAi). Using a genotype-phenotype association study, we further identified 5 new SNPs significantly associated with resistance in natural populations.

## Materials and methods

### Ethics statement

This study did not involve endangered or protected species, and was not conducted on protected lands. All collections were performed on public land, and no specific permits were required for mosquito field specimen collection.

### *Ae*. *albopictus* sample and transcriptome library preparation

#### Insecticide resistance bioassay

The overall study design is shown in S1 Fig. RNA-seq used deltamethrin-resistant and -susceptible *Ae*. *albopictus* mosquitoes. Immature *Ae*. *albopictus* (larvae and pupae) samples were collected in September 2014 in the Longgang district, Shenzhen, China (22.7210° N, 114.2469° E), in public areas such as streets, parks, and parking lots, from more than 50 different aquatic habitats, including discarded plastic containers, flower pots, and used tires. The field-collected larvae/pupae were reared to adulthood at 28°C, 70–80% relative humidity, with 16:8h light-dark photoperiods in the insectary of Southern Medical University. After emerging, 3-5-day old female adults were identified to species by morphology before resistance bioassay using the standard WHO insecticide susceptibility tube test with 0.05% deltamethrin. The 0.05% deltamethrin and control filter papers were purchased from the WHO reference laboratory in the School of Biological Sciences, Universiti Sains Malaysia (Penang, Malaysia). Mosquitoes that were alive after the 24 h recovery period were classified as resistant (R). Susceptible ones (S) were defined as those knocked-down within the first 20 min of the standard WHO insecticide susceptibility tube test and not able to fly 3 h after being moved to a recovery tube [33, 34]. Mosquitoes were carefully examined to ensure that the inability to fly was not due to loss of legs or wings. Mosquitoes were then preserved in RNA-later (Ambion, Carlsbad, CA, USA). All bioassays were conducted under a controlled environment at 28°C and 70–80% relative humidity in the insectary of Southern Medical University. All specimens were further confirmed for species identity using PCR as previously described [13].

#### RNA-seq library construction

RNA-seq library preparation and sequencing were carried out by HudsonAlpha Genomic Services Lab (Huntsville, AL, USA). A total of 24 cDNA libraries were prepared individually from 12 susceptible and 12 resistant mosquitoes. The main reason that we constructed libraries from individual mosquitoes was that this strategy enabled us to detect both transcriptome changes and SNPs associated with resistance using the RNA-seq data. This approach has advantages over the pooled mosquito approach, as the latter approach cannot detect SNPs with different frequencies between resistant and susceptible mosquitoes. Total RNA was extracted from 12 resistant and 12 susceptible female mosquito samples individually using Trizol reagent (Invitrogen, Carlsbad, CA, USA) according to the manufacturer’s protocol. Recombinant DNase I (Takara, Japan) was used to remove potential genomic DNA. RNA integrity (RNA Integrity Score 6.8) was evaluated using Nanodrop 2000 (Thermo Scientific, Delaware, ME, USA). RNA quality was analyzed on an Agilent 2100 Bio analyzer (Agilent, Palo Alto, CA, USA). Genomic DNA was extracted from mosquito legs using a Fast Tissue-to-PCR kit (Thermo fisher, Delaware, ME, USA) following manufacturer’s protocol.

The RNA-seq libraries were prepared with 500 μg of starting total RNA with an Illumina TruSeq RNA Sample Preparation Kit (Illumina, San Diego, CA), following the TruSeq protocol. The libraries were amplified with 15 cycles of PCR and contained TruSeq indexes within the adapters, specifically, indexes 1–4. Finally, amplified library yields were 30 μl of 19.8–21.4 ng/μl with an average length of ∼270 bp, indicating a concentration of 110–140 nM. KAPA Library Quantification Kits Illumina Platforms (Illumina, CA, US) were used for quantitation and dilution, and the libraries were sequenced on an Illumina HiSeq 2500 instrument with 100 bp paired end (PE) reads.

#### qRT-PCR validation of RNA-seq data

We analyzed 11 randomly selected, differentially expressed genes by quantitative real-time reverse transcription PCR (qRT-PCR). Specifically, cDNA was prepared using SuperScript III (Invitrogen, MA, USA) and random primers from pooled RNA of 4 resistant or 4 susceptible mosquitoes, and three biological replicates were used. The qRT-PCR reactions were performed in a 7300 FAST Real-Time PCR System (ABI, Foster City, CA, USA) and analyzed using the *rpS*7 (ribosomal protein 7) gene as internal control [35]. All the qPCR primers used in this study are shown in S2 Table. Expression levels were then calculated against control mosquitoes as a calibrator using the 2^-ΔΔCt^ method with CFX Manager Software (CFX Manager 3.1, BioRed, USA). The Pearson correlation coefficient was calculated between fold changes in transcript accumulation levels between resistant and susceptible mosquitoes, as obtained by qRT-PCR and RNA-seq, respectively.

### RNAi analysis

RNAi analysis used a laboratory-selected deltamethrin-resistant population (Lab-DR strain). Briefly, larvae from a laboratory Foshan strain of *Ae*. *albopictus* were bioassayed to determine the 50% lethal concentration (LC_50_), following the WHO standard method [34]. This bioassay measured larval mortality after 24 hr of exposure to 5 concentrations of deltamethrin, each concentration in three replicates. The Lab-DR strain was selected from the larvae of the Foshan strain, using 0.001 mg/L of deltamethrin) as the starting concentration, and gradual increments of deltamethrin concentrations for 10 generations. The 10^th^ generation selection used a 0.015 mg/L concentration of deltamethrin. The WHO insecticide susceptibility tube test on Lab-DR adults found an 89% mortality rate, indicating modest resistance.

RNAi analysis was conducted in both adult mosquitoes and larval mosquitoes with 3 highly differentially expressed genes. For adult mosquito RNAi, 3-day post-emergence Lab-DR mosquitoes was randomly selected and divided into control group or RNAi groups. For each of the three RNAi groups, 500ng siRNA duplex (siRNACYP6A8, siRNA01931, or siRNA00656) was microinjected into a female mosquito thorax (S2 Table). For the control group, a siRNA duplex lacking significant sequence homology to any genes in the *Ae*. *aegypti* genome, siRNA_C, was injected (S2 Table). All siRNAs were synthesized by IDT Technologies (Coralville, IA, USA). After the injection, mosquitoes were allowed to recover in a cup and maintained with 8% sucrose. An overall 16% mortality was observed 24 hr after the injection. Forty-eight hours after injection, gene knockdown efficiency was examined in 12 randomly selected individuals in each injection group using qRT-PCR, and its impact on mosquito resistance was determined using a standard WHO insecticide susceptibility tube test with 0.05% deltamethrin. For each gene, RNAi-treated female mosquitoes 48 hr after injection underwent the WHO insecticide susceptibility bioassay, with 20–25 mosquitoes per replicate for a total of 5 replicates per group [36]. Control group and RNAi groups were tested simultaneously.

For larval mosquito RNAi, we conducted oral RNAi following the method of Zhang *et al*. [37]. Briefly, each target gene siRNA and the siRNA_C described above (S2 Table) was mixed with Chitosan to form a Chitosan/siRNA nanoparticle. The nanoparticle was then mixed with cat food and agarose with green food dye, before feeding to 20 second-instar larvae in a petri dish with 100 ml water on a daily basis until the adult mosquitoes emerged. A total of 20 replicates with a total of 400 mosquito larvae were used for each target gene group and the control group. All larvae were successfully fed with the nanoparticles as evidenced by the green food dye in their guts. Three days after emergence, for each group 12 female adult mosquitoes were randomly selected to detect gene expression by qRT-PCR. Deltamethrin resistance bioassay was conducted using the standard WHO insecticide susceptibility tube test, with 20–25 mosquitoes per replicate for a total of 5 replicates per group.

### Genotype-phenotype association study

#### P450-related SNP identification

RNA-seq analysis identified 1,162 SNPs with a minimum frequency difference of 0.5 between the susceptible and resistant individuals. Because cytochrome P450 genes were considered most important to pyrethroid resistance, we aimed to identify potential SNPs associated with resistance within the P450 gene family. Cytochrome P450-related SNPs were selected using the following criteria: Mutation frequency differences between resistant and susceptible individuals that were studied by RNA-seq were at least 50%, and Fisher’s exact test *P* < 0.005 between the phenotyped individuals.

#### Genotype-phenotype association analysis

To establish an association between SNP and phenotypic resistance in natural mosquito populations, we screened 1,350 female adults from Shenzhen, southern China, for deltamethrin resistance using the standard WHO insecticide susceptibility tube test, yielding 115 resistant mosquitoes. Randomly-selected resistant mosquitoes (n = 70) and susceptible ones (n = 70) were genotyped at 7 candidate genes to cover 9 resistance-associated SNPs identified through the above RNA-seq analysis. Primers used for SNP genotyping are shown in S2 Table. PCR products were purified with ExoSAP-IT (USB, Cleveland, Ohio, USA) according to the manufacturer’s protocol and directly sequenced by Genewiz Inc. (South Plainfield, NJ, USA).

#### *Kdr* mutation detection in Lab-DR strain adults and susceptible Foshan strain

Randomly selected female adult mosquitoes (n = 30) from Lab-DR and from the susceptible Foshan strain (n = 30) were genotyped for *kdr* mutations at the L1534 locus. Portions of domains III of the VGSC gene were amplified, following protocols and primers developed by Kasai et al. [38]. PCR products were sequenced by TsingKe Inc. (Beijing, China). The sequences were analyzed using BioEdit (http://www.mbio.ncsu.edu/BioEdit/bioedit.html).

### Data analysis

#### RNA-seq data analysis

Sequenced reads were assigned to each sample, and adaptors were removed. Read quality for each sample was checked using FastQC. Reads with quality scores less than 20 and lengths below 30 bp were all trimmed. The resulting high-quality sequences were used in subsequent assembly. Clean reads were used as input in all the assemblers in CLC Genomics Workbench (version 8.5.1). To identify differentially expressed transcripts, reads from resistant and susceptible conditions were mapped to the *Ae*. *albopictus* reference genome, available from Vector Base database, using the transcriptomic analysis module, with the following parameters: mismatches = 2, minimum fraction length = 0.9, minimum fraction similarity = 0.8, and maximum hits per read = 10. Gene expression levels were normalized as reads per kilobase of exon model per million mapped reads (RPKM). Theoretical values were obtained from means of RPKM values for each group. Transcripts with absolute fold-change values > 2.0 and FDR corrected P-values < 0.05 were included in the gene ontology (GO) and Kyoto Encyclopedia of Genes and Genomes (KEGG) pathways enrichment analyses. The differentially regulated genes were analyzed for gene ontology (GO) using the vector base *Ae*. *albopictus* assembly P3 database and categorized based on GO terms (level 2) for biological processes, molecular functions, and cellular components, as well as by genes up- or down-regulated in association with KEGG pathways.

SNP detection was processed for RNA-seq data obtained for individual resistant and susceptible mosquitoes with CLC Genomic Workbench, with the following parameters: Ignore positions with coverage > 100,000, ignore broken pairs, ignore non-specific match reads, with minimum read length = 20, minimum coverage = 10, minimum count = 2, minimum frequency = 35%, neighborhood radium = 5, minimum central quality = 20, minimum neighborhood quality = 15, relative read direction significance>1%, read position filter significance>1%. The site with substitution frequency above 5% was considered as a single nucleotide variant (iSNV). SNP_tool from MS-Excel was adopted to calculate the odds ratio (OR), chi-square analysis between resistant and susceptible individuals. Statistically significant SNPs were selected with OR > 2 and *P* < 0.005. We expect RNA-seq data to include population variations and to reflect differential expression of transcripts because the mosquitoes we used were collected from the field and our experimental design compared mosquitoes with two distinct phenotypes (resistant and susceptible).

#### RNAi data analysis

A *t-*test was used to compare expression levels between experimental groups, and the χ^2^ test was used to compare 24 h mortality rates among different treatments. If mortality exceeded 20% in the control group, the replicate was excluded from the analysis. If mortality in the controls was < 20%, the mortality rate with the particular treatment was corrected using Abbott’s formula [39].

#### Genotype-phenotype association data analysis

The sequences were aligned and analyzed using BioEdit (http://www.mbio.ncsu.edu/BioEdit/bioedit.html) and Codon code (http://www.codoncode.com/). The odds ratio was calculated to examine the association between SNP and resistance, and Fisher’s exact test was performed to determine the statistical significance.

### Data accessibility

All RNA-seq data generated in this study are available under NCBI BioProject number PRJNA475859.

## Results

### RNA-seq and quality control

Twenty-four cDNA libraries were constructed individually for deep sequencing on an Illumina-Solexa HiSeq 2500 platform. Raw read quality control was carried out via base composition analysis and base sequence quality analysis. A total of 674,503,592 and 612,512,034 paired-ends reads from resistant and susceptible conditions, respectively, were obtained. A summary of filtered paired-end reads is shown in Table 1. An average of 56,208,633 reads in resistant individuals and 51,042,670 in susceptible individuals were mapped to the *Ae*. *albopictus* reference genome. Gene coverage statistics for resistant and susceptible individuals showed that most of the genes (84% and 77%, respectively) reached coverage of 90–100%.

**Table 1:** Summary of filtered RNA-seq data for deltamethrin-resistant and -susceptible *Aedes albopictus* individuals.

	Resistant	Susceptible
**Individual libraries**	12	12
**Total reads**	674,503,592	612,512,034
**Average reads**	56,208,633	51,042,670
**Standard deviation**	13,126,099	11,991,432
**Maximum reads**	87,920,874	82,427,942
**Minimum reads**	36,155,374	32,821,820
Average gene coverage of 90–100%*	84% (12,632)	77% (12,759)

* Gene coverage was calculated as the ratio of the base number a gene covered by unique mapping reads to the total base number of the coding region in the gene. Average gene coverage of 90–100% denotes that 90–100% of a gene was covered by unique mapping reads.

### Gene ontology enrichment analysis and pathway enrichment analysis of DEGs

**Fig 1:**
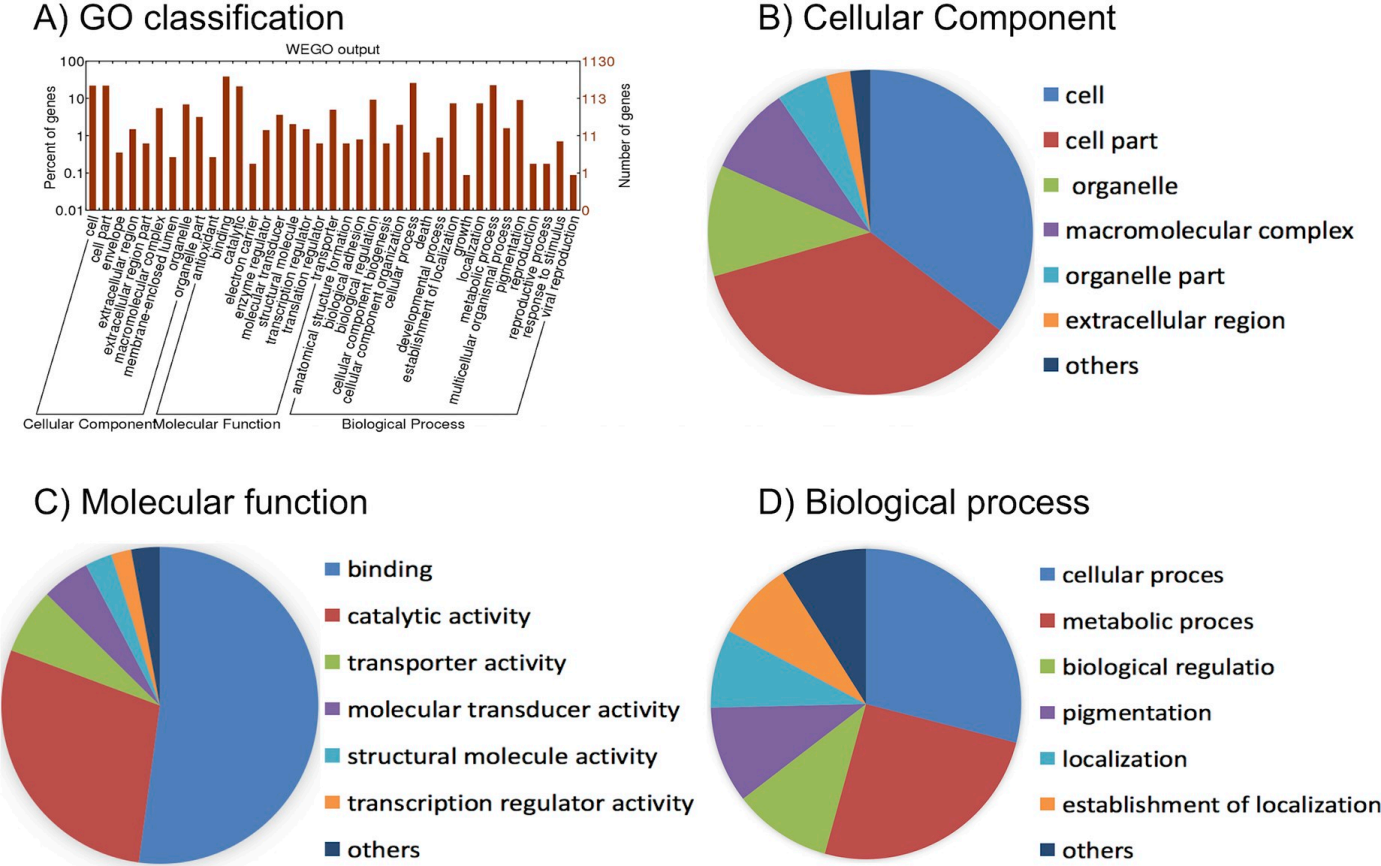
Gene Ontology (GO) analysis of differentially expressed genes (DEGs) of *Aedes albopictus* transcriptome annotated to UniGene cDNA reference data. Analysis was performed with Blast2GO-PRO. A) Histogram of *Ae*. *albopictus* differentially expressed sequences GO classification. Three main ontologies of GO (biological process, cellular component, and molecular function) are shown in the x-axis. The left y-axis indicates the percentage of total genes, and the right y-axis is the number of genes in each category. B) Distribution of transcripts in category of cellular components. C) Distribution of transcripts in category of molecular functions. D) Distribution of transcripts in category of biological processes.

All unique matched reads from resistant and susceptible individuals were assembled into 20,091 transcripts in a total of 11,090 genes. From these genes, 1,130 genes were significantly differentially expressed genes (DEGs) when using the criteria of FPKM ratio of each phenotype >2 (in either direction), *P* < 0.0001, and FDR < 0.05. Among them, 874 genes (77.3%) were up-regulated and 256 genes (22.7%) were down-regulated in the resistant mosquitoes. These DEGs included P450s, cuticle proteins, UDP-glucuronosyltransferases, lipases, serine proteases, heat shock proteins, esterase, peptidases, ATP-binding cassette transporters, and others (Fig 1A).

All DEGs were classified into three main GO categories: biological processes, cellular components, and molecular functions (Fig 1). For cellular components, genes involved in cells (GO: 0005623, 249 out of 705 DEGs, or 35.3%) and cell parts (GO: 0044464, 249 out of 705 DEGs, 35.3%) were the most abundant (Fig 1B). As for molecular functions, binding (GO: 0005488, 435 out of 838 DEGs, 51.9%) and catalytic activity (GO: 0003824, 238 out of 838 DEGs, 28.4%) were the most highly represented categories (Fig 1C). For biological processes, the categories most represented were cellular processes (GO: 0009987, 294 out of 1,013 DEGs, or 29.0%) and metabolic processes (GO: 0008152, 256 out of 1,013 DEGs, 25.3%) (Fig 1D).

A total of 694 DEGs were mapped into 309 KEGG pathways. The largest category was metabolic pathways, containing 92 annotated DEGs (20.6%), followed by biosynthesis of secondary metabolites, with 36 DEGs (8.1%). In general, the most represented GO and KEGG categories were “metabolic process” and “metabolic pathway,” which were widely acknowledged to be involved in insecticide metabolism [40,41]. A total of 11 DEGs belonging to the P450 family or GST which were known to be related to insecticide detoxification are shown in Table 2. We did not find any significant DE genes related to metabolic inhibitors or cuticle protein genes.

**Table 2:** Differentially expressed genes involved in detoxification in deltamethrin resistance and susceptible *Aedes albopictus*.

Transcript ID	Chromosome	Region	R FPKM mean	S FPKM mean	R_mean /S_mean	T-Test *P*-value	GenBank	Gene name
**CCG005550.1**	lcl|scaffold1478	complement (165639..167290)	6.90	1.69	4.07	0.0063	tca:664295	*CYP9Z4*
**CCG019182.1**	lcl|scaffold5098	114116..115695	22.49	7.26	3.10	0.0602	dme:Dmel_CG12800	*CYP6D4*
**CCG015447.1**	lcl|scaffold3790	complement (10696..11999)	1.22	0.41	2.95	0.0154	tca:655001	*CYPIVC3*
**CCG010715.1**	lcl|scaffold242	complement (13540..15127)	0.13	0.05	2.57	0.2129	dme:Dmel_CG12800	*CYP6D4*
**CCG015756.1**	lcl|scaffold3898	16591..18932	7.33	3.03	2.42	0.0294	dme:Dmel_CG8864	*CYP28A5*
**CCG012909.1**	lcl|scaffold29929	654..1879	11.35	4.76	2.39	0.0029	dme:Dmel_CG3656	*CYP4D1*
**CCG012356.1**	lcl|scaffold2844_2	17180..18196	1.94	0.85	2.28	0.1840	tca:664295	*CYP9Z4*
**CCG024122.1**	lcl|scaffold7278	complement (5503..7082)	541.31	243.22	2.23	0.1730	dme:Dmel_CG9438	*CYP6A2*
**CCG018948.1**	lcl|scaffold501	270699..272928	46.34	21.00	2.21	0.0469	dme:Dmel_CG10248	*CYP6A8*
**CCG007720.1**	lcl|scaffold17647	11732..12808	2.84	1.31	2.18	0.0684	dme:Dmel_CG4373	*CYP6D2*
**CCG018086.1**	lcl|scaffold4704	29466..30259	7.78	2.09	3.72	0.2003	aga:AgaP_AGAP009195	*GSTE1*

R: Resistance, S: Susceptible

#### SNP identification

A total of 267,469 non-synonymous mutation sites were identified; among them, 1,418 polymorphisms differed significantly in frequencies between resistant and susceptible mosquitoes. These included 1,162 SNPs (81.9%), 162 multiple nucleotide polymorphisms (MNPs) (13.9%), 37 insertions (3.2%), and 39 deletions (3.4%). Among the 1,162 SNPs detected, 766 SNPs (54.1%) were mapped to the genes with annotation. Among the 766 SNPs with annotation, 9 SNPs belong to 7 P450 genes and 3 GSTs (Table 3). In the *kdr* gene, mutations from wildtype TTC (Phe) to TTG (Leu), or to TCC (Ser) at codon 1534 were detected, with an F1534S mutation frequency of 29.1% in resistant individuals and 16.7% in susceptible individuals.

**Table 3:** Nucleotide polymorphism of genes belonging to metabolic P450 gene family and Glutathione-S-Transferase (GST) significantly expressed between deltamethrin-resistant and -susceptible *Ae*. *albopictus* individuals.

Transcript ID	Gene annotation	Coding region change	Amino acid change	Resistant population		Susceptible population		OR (95%CI)	*P*
				No. homozygote^a^	No. heterozygote^b^	No. homozygote^a^	No. heterozygote^b^		
**CCG018947.1**	*CYP6A8*	678A>C	Arg226Ser	8	2	2	1	11.4 (3.0–44.0)	<0.0001
		836A>G	Asp279Gly	9	0	0	1	69.0 (7.6–625.9)	<0.0001
**CCG009469.1**	*CYP4G1*	740T>C	Ile247Thr	8	0	0	0	NA	
**CCG006037.1**	*CYP9B2*	524C>A	Pro175Gln	7	5	2	1	14.4 (3.6–58.2)	<0.0005
**CCG006695.1**	*CYP9B2*	2629C>T	His877Tyr	7	3	2	0	12.1 (3.0–48.7)	<0.005
**CCG015439.1**	*CYP6A8*	360G>A	Met120Ile	9	0	3	0	9 (2.5–34.7)	<0.005
**CCG006036.1**	*CYP9B1*	845T>G	Leu282Arg	7	3	2	1	9.2 (2.5–34.6)	<0.005
**CCG027556.1**	AGAP009246-PA; cytochrome P450	1161A>C	Glu387Asp	9	2	4	0	10 (2.5–39.3)	<0.005
**CCG011108.1**	*CYP1A1*	634T>A	Cys212Ser	8	3	3	1	9.2 (2.5–34.6)	<0.005
**CCG007313.1**	*GST theta K01800*	314A>C	Glu105Ala	11	1	5	1	27.2(3.1–235.0)	<0.005
**CCG007571.1**	*GST theta K00799*	361G>A	Ala121Thr	6	6	2	1	11.4 (3.0–44.0)	<0.005
**CCG011234.1**	*GST theta K00799*	1328G>A	Arg443Lys	8	2	1	3	11.4 (3.0–44.0)	<0.005

R: resistant, S: susceptible, NA: not applicable. P: Fisher Exact Probability Test.

^a^ Number of individuals with homozygous SNP genotype. For example, for gene *CYP6A8* SNP 678A>C, there were 8 individuals with CC genotype out of the 12 resistant individuals sequenced.

^b^ Number of individuals with heterozygous SNP genotype. For example, for gene *CYP6A8* SNP 678A>C, there were 2 individuals with AC genotype out of the 12 resistant individuals sequenced.

### Comparison of transcription level between RNA-seq and qRT-PCR

**Fig 2:**
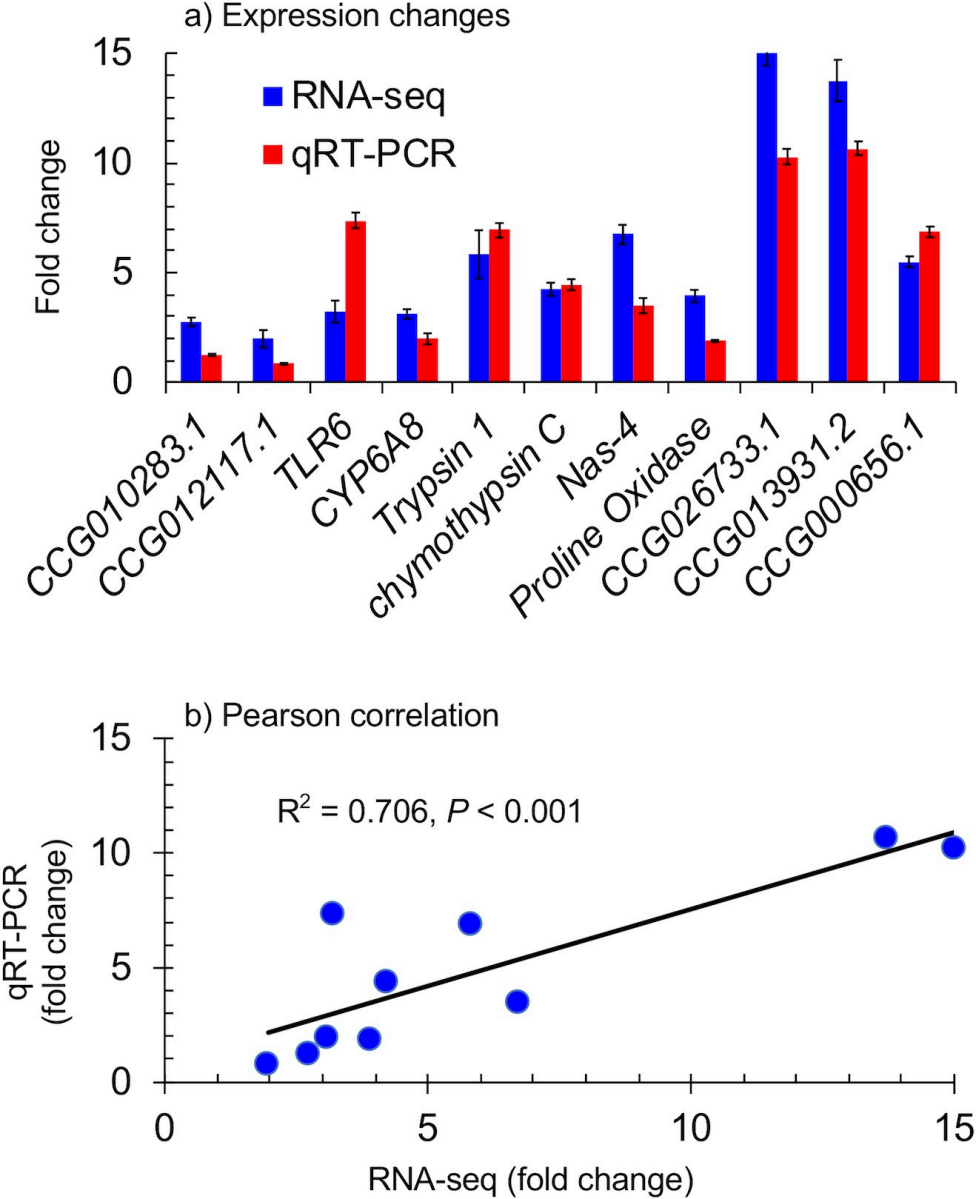
Comparison of expression value measured by RNA-seq and qRT-PCR. A) Fold change of expression value in the resistant individuals relative to the susceptible individuals, as measured by RNA-seq and qRT-PCR in 11 selected genes. B) Pearson correlation between fold changes in gene expression between resistant and susceptible mosquitoes, as determined by qRT-PCR and RNA-seq (*P*<0.05).

We used qRT-PCR to validate the expression level obtained from RNA-seq. As shown in Fig 2, a highly significant correlation was found between the two methods (R^2^ = 0.67), demonstrating the validity of the RNA-seq method for expression quantitation.

### RNAi analysis indicated *CYP6A8* (CCG018948), CCG013931.2, and CCG000656.1 played roles in deltamethrin resistance of *Ae*. *albopictus*

*CYP6A8* (CCG018948), CCG013931.2, and CCG000656.1 were selected for RNAi analysis based on expression differences between resistant and susceptible mosquitoes. The RNA-seq analysis indicated that the expression of *CYP6A8* was 3.1-fold higher in the resistant samples than in the susceptible ones, and CCG013931.2 and CCG000656.1 were 13.7 and 5.5-fold higher as evidenced from the (Fig 2). For microinjection RNAi in adult mosquitoes, the *CYP6A8* expression level was reduced by 52.8% by siRNACYP6A8, compared with the control (*P* < 0.01) (Table 4). Similarly, CCG013931.2 expression level was reduced by 66.3% (*P* < 0.01), and by 71.4% for CCG000656.1 (*P* < 0.01). The resistance bioassay found 100% mortality in RNAi treated groups for the three genes whereas a 90.1% mortality rate was found in the control group (*P* < 0.01) (S3 Table). The effect of adult mosquito RNAi using the microinjection method on mosquito knockdown time is shown in S2 Fig. Mosquitoes injected with siRNA01931 exhibited significantly shorter KDT_50_ time (S3 Table).

**Table 4:** Effects of RNAi on the expression of three candidate resistance genes in *Aedes albopictus*. Microinjection and oral delivery RNAi were used.

	Microinjection		Oral delivery	
Gene	Control^a^	RNAi^b^	Control^a^	RNAi^b^
*CYP6A8*	1.000 (0.045)	0.472 (0.063)**	1.000 (0.017)	0.481 (0.016)**
*CCG013931*.*2*	1.000 (0.079)	0.337 (0.062)**	1.000 (0.026)	0.465 (0.057)**
*CCG000656*.*1*	1.000 (0.054)	0.286 (0.031)**	1.000 (0.004)	0.520 (0.012)**

^a^ Control refers to *Ae*. *albopictus* Lab-DR strain mosquitoes treated with a siRNA duplex lacking significant sequence homology to any genes in the *Ae*. *aegypti* genome.

^b^ RNAi: siRNA-treated group

**, *P* < 0.01 for comparison between RNAi group and control group.

Oral feeding of siRNA nanoparticles in larvae indicated a similar gene expression knockdown effect compared to the microinjection group. The *CYP6A8* expression level was reduced by 51.9% compared with the control (*P* < 0.01), by 53.5% for CCG013931.2 (*P* < 0.01), and by 48.0% for CCG000656.1 (*P* < 0.01) (Table 4). Gene expression knockdown corresponded to increased mortality rate in the RNAi-treated groups. Mortality rate was 90.5% in the control group whereas the RNAi-treated groups showed 100% mortality rate (*P* < 0.01) (S4 Table). The mosquito knockdown time in insecticide resistance bioassay found a significantly shorter average knockdown time in RNAi treated groups than the control group (S2 Fig).

### Frequency of *kdr* mutations in Lab-DR resistant strain and susceptible Foshan strain

Non-synonymous mutation was detected at codon 1534 in the selected Lab-DR resistant strain adults but not in the susceptible Foshan strain. At codon 1534, a change from wildtype codon TTC (Phe) to TCC (Ser) was detected in 14 out of the 30 samples, of which all were heterozygotes. The L1534S mutation frequency was 23.3% in the selected Lab-DR resistance strain.

### Association between SNPs in P450 gene family and pyrethroid resistance in natural mosquito population

We examined 9 SNPs within the 7 P450 genes identified from the RNA-seq analysis to determine SNP genotypes’ association with deltamethrin resistance using 70 randomly selected resistant mosquitoes and 70 susceptible mosquitoes (Table 5). Among the 9 SNPs examined, 5 (Arg226Ser in *CYP6A8*, Pro175Gln and His877Tyr in *CYP9B2*, Met120Ile in *CYP6A8*, and Cys212Ser in *CYP1A1*) showed significant associations with resistance.

**Table 5:** Association between selected P450 gene SNP frequency and deltamethrin resistance in natural *Aedes albopictus* populations.

Gene	Transcript ID	Amino acid change	Phenotype	N	Genotypes			Odds ratio (95%CI)	*P*
annotation					Homozygous mutation	Heterozygous mutation	Homozygous wild		
***CYP6A8***	CCG018947.1	Arg226Ser	R	43	7	6	30	19.09 (2.49–147)	< 0.0001
			S	32	0	1	31		
		Asp279Gly	R	65	5	18	42	1.84 (0.75–4.52)	0.178
			S	27	0	7	20		
***CYP9B2***	CCG006037.1	Pro175Gln	R	63	20	11	32	2.95 (1.43–6.06)	<0.005
			S	32	4	4	24		
***CYP9B2***	CCG006695.1	His877Tyr	R	41	26	9	6	5.05 (2.38–10.7)	< 0.0001
			S	26	8	3	15		
***CYP6A8***	CCG015439.1	Met120Ile	R	51	4	2	45	6.85 (0.86–54.8)	0.037
			S	32	0	1	31		
***CYP9B1***	CCG006036.1	Leu282Arg	R	57	2	3	52	NA	NA
			S	32	0	0	32		
***CYP1A1***	CCG011108.1	Cys212Ser	R	62	55	5	2	55.37 (21.97–140)	<0.0001
			S	32	5	2	25		

NA: not applicable. Odds ratio measures the association between SNP genotype and resistance.

## Discussion

In the present study, we used the RNA-seq technique to study the transcriptional profile of deltamethrin-resistant and -susceptible *Ae*. *albopictus* mosquitoes. The individual mosquito-based sampling strategy used in this study is distinct from the commonly used pooled sampling strategy, and this new strategy enables us to simultaneously identify differentially expressed genes and differential SNPs that may be associated with pyrethroid resistance. We identified a total of 1,130 differentially expressed genes, including 874 up-regulated and 256 down-regulated genes. A total of 1,162 SNPs with large frequency differences between resistant and susceptible mosquitoes was identified. Using genotype-phenotype association analysis of natural mosquito populations, we identified 5 SNPs in the P450 gene family that are significantly associated with resistance. RNAi in adult mosquitoes through microinjection and larval mosquitoes through oral feeding confirmed three highly differentially expressed genes’ roles in resistance.

In our data analysis, most of the DEGs are related to metabolic pathways involving insecticide absorption, especially genes coding for cytochrome P450 monooxygenase (P450) and cytochrome C oxidase. The highly expressed DEGs in resistant individuals included specific P450 genes, such as *CYP6A8*, *CYP9Z4*, and *CYP6D4*, that were identified in other insects, such as *Musca domestica*, *An*. *gambiae*, *Cx*. *quinquefasciatus*, and *An*. *sinensis* [42–45]. We also detected one glutathione S-transferase (GST) gene (*GSTE1*, AGAP009195-PA) that showed increased expression in resistant mosquitoes, which was consistent with previous studies [15,34,46]. In addition to the up-regulated *CYP* genes, it was interesting to notice that there were several down-regulated *CYP* genes, including *CYP301A1*, *CYP4AC2*, and *CYP4V2*. Several *GST* genes were found to be down-regulated (*GSTE1* and *GSTE5*), which apparently contradicts the current knowledge on *GST*. GST enzymes have been shown to metabolize insecticides by facilitating reductive dehydrochlorination or by conjugation reactions with reduced glutathione, to produce water-soluble metabolites that are more readily excreted. The increased GST expression in resistant mosquitoes contributes to the removal of toxic oxygen-free radical species produced through the action of pesticides [47]. Thus, the current study’s reverse finding indicates that the GST enzyme may confer insecticide resistance in a more complex way. One explanation for decreased expression in the resistant individuals is that the decrease in gene expression may be due to reactions to various endogenous and exogenous compounds, or alternatively may represent a pathophysiological signal [48,49]. Such a GST gene down-regulation observed in resistant *Ae*. *albopictus* individuals was also found in other mosquito species: *Cx*. *quinquefasciatus* and *An*. *sinensis* [50,51]. These findings suggest complex associations between metabolic genes’ expression changes and insecticide resistance.

An RNA interference assay was conducted to verify the function of candidate genes identified from RNA-seq analysis. We used two RNAi methods: the classic microinjection-based and oral feeding RNAi. We demonstrated that both methods led to >50% reduction in the expression of 3 candidate genes, and that RNAi treatment altered the resistance phenotype. Our results indicated that these 3 genes each may played a role in deltamethrin resistance regulation, however the impact of these genes on insecticide resistance at the organismal level needs further determination. One major disadvantage of the microinjection-based RNAi method is that siRNA injection led to substantial mortality in adult mosquitoes. In the control, we observed 16% mosquito mortality, likely due to mechanical injury associated with microinjection. The oral RNAi showed less than 1% mortality from the oral delivery process, consistent with oral delivery RNAi methods reported by other studies [52]. Another advantage of oral delivery RNAi is the relative ease of laboratory operation, without the need for microinjection, and subsequently a large number of RNAi-treated mosquitoes can be used for the resistance bioassay. Therefore, oral RNAi may be considered in future studies.

Using a genotype-phenotype association study with field mosquito samples from southern China, we identified 5 SNPs within the *CYP* gene family that were significantly associated with pyrethroid resistance. In the SNP analysis, we also found higher F1534S mutations in the *kdr* gene among the 12 resistant individuals than in the 12 susceptible individuals (29.1% vs. 16.7%). The L1534S mutation frequency was 23.3% in the selected resistant strain but 0 in the laboratory susceptible colony. Therefore, the impact of mutations in the *kdr* gene on pyrethroid resistance is contingent on the mosquitoes’ genetic background. Mutations in other genes and expression of detoxification genes need to be considered in developing predictive biomarkers for resistance.

Our study has several limitations. First, only 12 resistant and 12 susceptible individuals were subjected to RNA-seq analysis. RNA-seq using a large sample size would detect more genetic variants with small effects on resistance. Secondly, the laboratory resistant population used for RNAi functional studies was not highly resistant to deltamethrin, which lowered the ability to detect candidate genes’ phenotypic impact. Nonetheless, the present study reveals the major genetic variants associated with pyrethroid resistance in *Ae*. *albopictus*.

## Conclusions

By comparing the expression profile of deltamethrin-resistant and -susceptible wild mosquitoes from China, we identified 3 candidate genes with increased expression in resistant *Ae*. *albopictus*. We determined that if interfered with, the mosquito would change phenotype status from resistant to susceptible, suggesting that these genes play a role in insecticide resistance. We also identified 5 SNPs in 4 P450 genes that were significantly associated with insecticide resistance. Overall, this study demonstrates the power of individually based transcriptome profiling, with the combination of RNAi and genotype-phenotype association analysis, in research on resistance mechanisms. That is, both differentially expressed genes and SNPs associated with pyrethroid resistance were identified in *Ae*. *albopictus* mosquitoes. Findings from this study provided candidate resistance genes and SNPs for future functional verification using the new gene editing techniques such as CRISPR/Cas9.

## Supporting information

S1 Fig Flowchart of research design. (PDF)

S2 Fig Effect of RNAi on the insecticide knockdown phenotype in *Aedes albopictus*. Insecticide knockdown phenotype was the time taken for a female mosquito to be knocked down in the standard WHO insecticide susceptibility tube test. a) Adult RNAi with *CYP6A8* microinjection; b) adult RNAi with CCG013931.2 microinjection; c) adult RNAi with CCG000656.1 microinjection; d) larval RNAi with *CYP6A8* oral delivery; e) larval RNAi with CCG013931.2 oral delivery; and f) larval RNAi with CCG000656.1 oral delivery. The mean knockdown time was compared between the specific RNAi group and control group, and appropriate statistic and *P* values are shown.
(PDF)

S1 Table A list of gene names, functions, and primers used in qRT-PCR for RNA-seq validation and siRNA sequence for *Aedes albopictus* RNA interference assay. (XLSX)

S2 Table PCR primer sequences for *CYP* gene polymorphism analysis in natural *Aedes albopictus* populations. (DOCX)

S3 Table Knockdown time and mortality rate of *Aedes albopictus* with RNAi delivered by the microinjection method. (DOCX)

S4 Table Knockdown time and mortality rate of *Aedes albopictus* with orally delivered RNAi. (DOCX)
