# Supplementary figures and images for: Comparative transcriptome analysis and RNA interference reveal CYP6A8 and SNPs related to pyrethroid resistance in Aedes albopictus
Source: PLoS Negl Trop Dis. 2018 Nov 12;12(11):e0006828. doi: 10.1371/journal.pntd.0006828 (PMC6258463; doi:10.1371/journal.pntd.0006828)

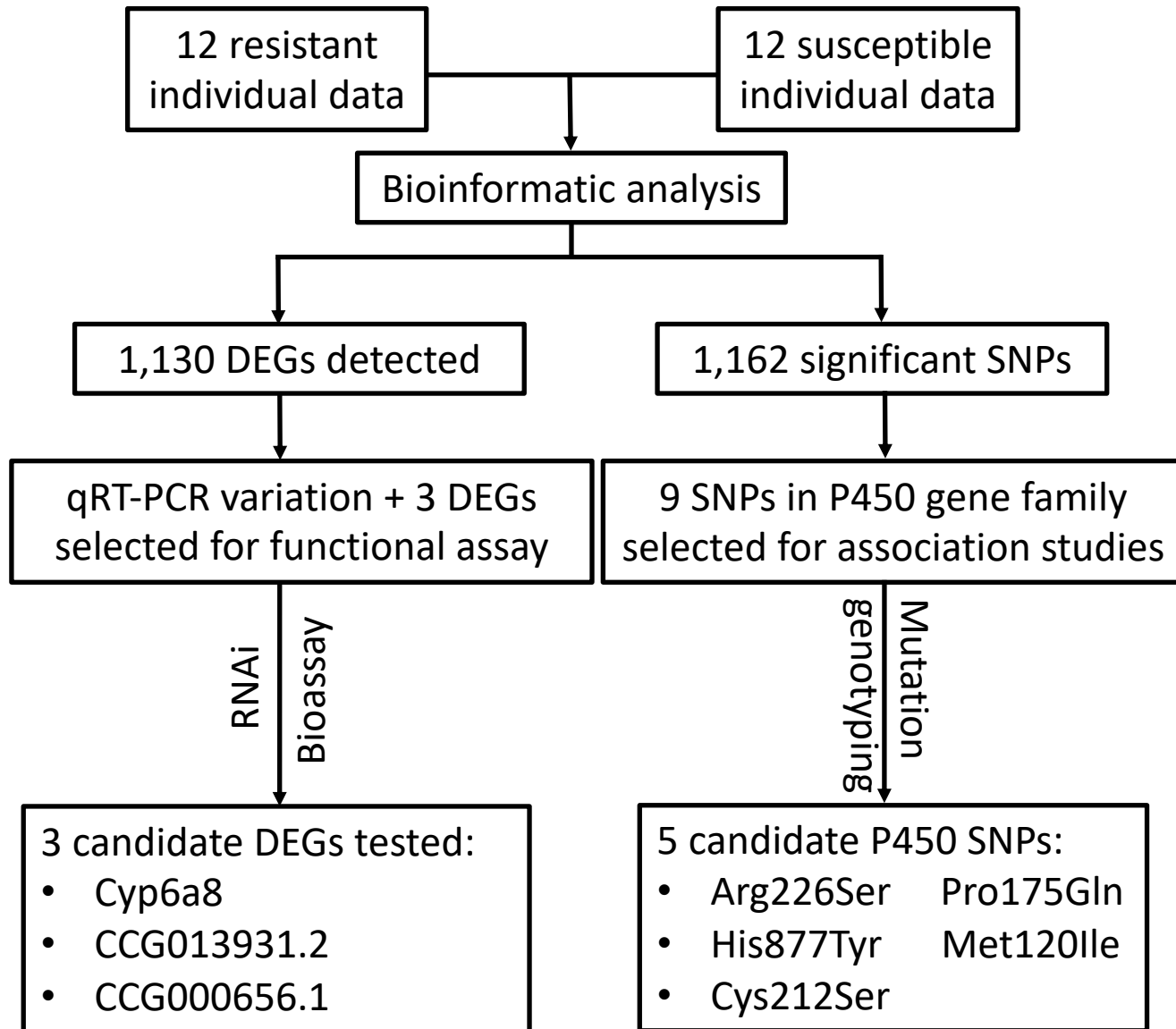

Supplement: S1 Fig — (PDF) [file pntd.0006828.s001.pdf]

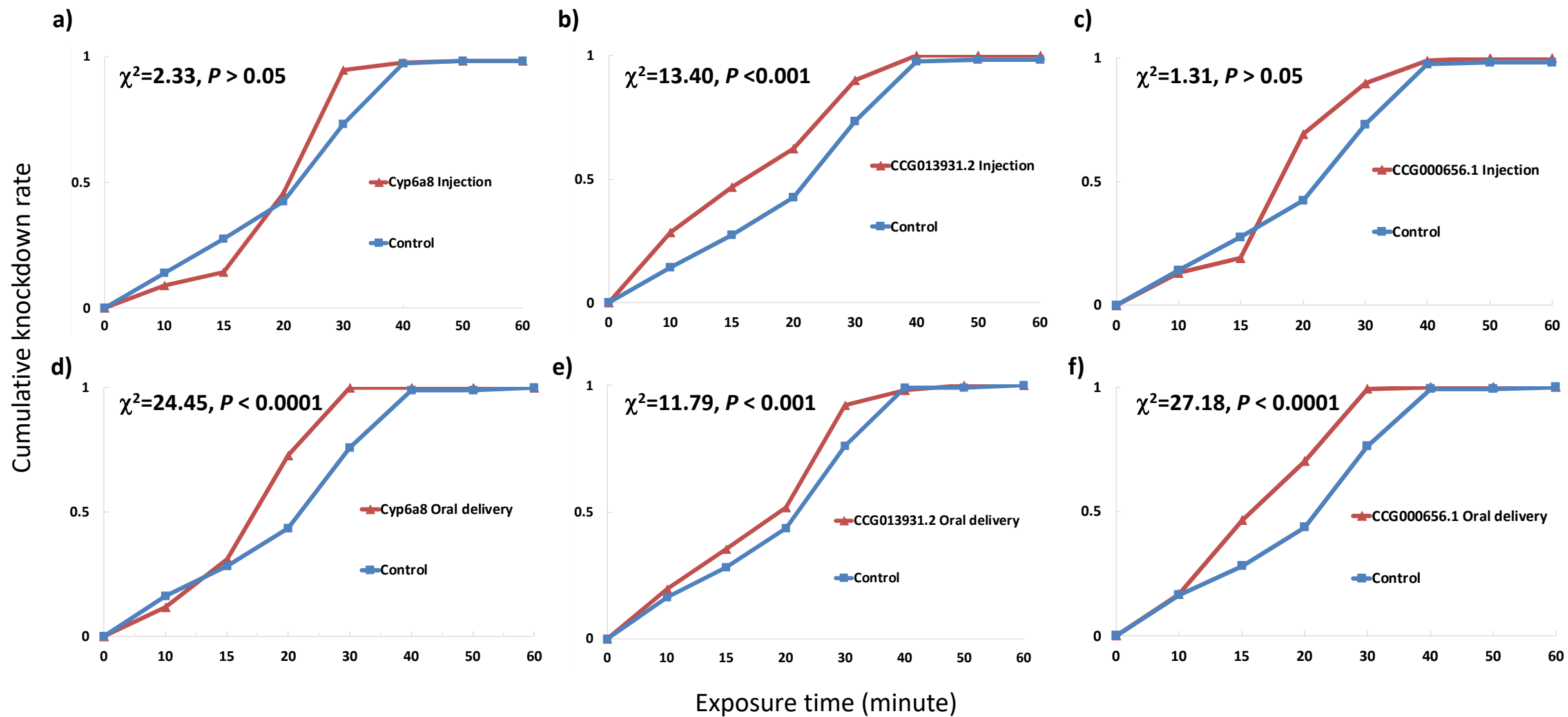

Supplement: S2 Fig — Insecticide knockdown phenotype was the time taken for a female mosquito to be knocked down in the standard WHO insecticide susceptibility tube test. a) Adult RNAi with CYP6A8 microinjection; b) adult RNAi with CCG013931.2 microinjection; c) adult RNAi with CCG000656.1 microinjection; d) larval RNAi with CYP6A8 oral delivery; e) larval RNAi with CCG013931.2 oral delivery; and f) larval RNAi with CCG000656.1 oral delivery. The mean knockdown time was compared between the specific RNAi group and control group, and appropriate statistic and P values are shown. (PDF) [file pntd.0006828.s002.pdf]
