# Supplementary material for: Comparative transcriptome analysis and RNA interference reveal CYP6A8 and SNPs related to pyrethroid resistance in Aedes albopictus
Source: PLoS Negl Trop Dis. 2018 Nov 12;12(11):e0006828. doi: 10.1371/journal.pntd.0006828 (PMC6258463; doi:10.1371/journal.pntd.0006828)
Supplement: S2 Table — (DOCX) [file pntd.0006828.s004.docx]

**S3 Table. PCR primer sequences for *CYP* genetic association study in natural *Aedes albopictus* populations.**

| Transcript ID | Scaffold position | Gene annotation | Primer name | sequence | bp |
| --- | --- | --- | --- | --- | --- |
| CCG018947.1 | scaffold501 | Cyp6a8 | C1-F1 | CACGTCTGGCAAGATGAAGA | 352 |
|  |  |  | C1-R1 | CGATGGTGCTCTCCACTACA |  |
|  |  |  | C2-F1 | AAGCAGGAGAGCGAGTTTGA | 423 |
|  |  |  | C2-R1 | TTTCAAATCCGGCCAAGTAG |  |
| CCG009469.1 | scaffold211 | Cyp4g1 | C3-F1 | GATAACAGTTTGGCCGTGGT | 434 |
|  |  |  | C3-R1 | AAGTGAGCCCTGCGAGATAA |  |
| CCG006037.1 | scaffold15348 | Cyp9b2 | C4-F1 | CCAACATTCCGATGTACGTG | 490 |
|  |  |  | C4-R1 | TAACCGGCCCATCAAAGTAG |  |
| CCG006695.1 | scaffold162 | Cyp9b2 | C5-F1 | AGCGATTGAGTGTGTTGCTG | 456 |
|  |  |  | C5-R1 | TGCTTTCCCTGTAGCGATTC |  |
| CCG015439.1 | scaffold3789 | Cyp6a8 | C6-F1 | CACTGGCCGTTTACCTGTTT | 475 |
|  |  |  | C6-R1 | TTCAAATTCGCTCTCCTGCT |  |
| CCG006036.1 | scaffold15348 | Cyp9b1 | C7-F1 | GATCATCAAGGACGCTGTGA | 230 |
|  |  |  | C7-R1 | CCTGCCAGGAAGAATACCAA |  |
| CCG027556.1 | scaffold924 | cytochrome P450, family 4, subfamily V | C8-F1 | CAGGCAGTTCAAGACCGAAT | 445 |
|  |  |  | C8-R1 | ACCGGAAGTACTCGGAACCT |  |
| CCG011108.1 | scaffold2512 | Cyp1A1 | C9-F1 | CTTCGCCAACCATCCATACT | 396 |
|  |  |  | C9-R1 | CATCCACTTGGGGAACAGAT |  |
