# Supplementary material for: Comparative transcriptome analysis and RNA interference reveal CYP6A8 and SNPs related to pyrethroid resistance in Aedes albopictus
Source: PLoS Negl Trop Dis. 2018 Nov 12;12(11):e0006828. doi: 10.1371/journal.pntd.0006828 (PMC6258463; doi:10.1371/journal.pntd.0006828)
Supplement: S3 Table — (DOCX) [file pntd.0006828.s005.docx]

**S5 Table. Knockdown time and mortality rate of *Aedes albopictus* with RNAi delivered by the microinjection method.**

| Group | Gene targeted by RNAi | n | KDT_50_ (95% CI) | KRR_50_^b^ | 24h corrected mortality rate |
| --- | --- | --- | --- | --- | --- |
| RNAi | *Cyp6a8* | 130 | 21.9 (20.2-22.8) ^ns^ | 1.9 (1.7-2.1) ^ns^ | 100% ** |
|  | *CCG013931.2* | 120 | 16.1 (14.7-17.5) * | 1.4 (1.3-1.5) * | 100% ** |
|  | *CCG000656.1* | 130 | 19.0 (16.4-21.8) ^ns^ | 1.6 (1.4-1.8) ^ns^ | 100% ** |
| Control^a^ |  | 120 | 22.3 (17.2-27.1) | 2.0 (1.6-2.4) | 90.1% |

Note: Mortality rate was measured using the standard WHO insecticide susceptibility tube test against 0.05% deltamethrin.

^a^ Control refers to *Ae. albopictus* Lab-DR strain mosquitoes injected with a siRNA duplex lacking significant sequence homology to any genes in the *Ae. aegypti* genome.

^b^ KRR_50_ was calculated as the ratio of KDT_50_ of the treatment group to KDT_50_ of the susceptible Foshan mosquito group.

**P* < 0.05; and ** *P* < 0.01 for comparison between RNAi group and control group; *ns* indicates no statistical significance between RNAi group and control group.
