# Supplementary material for: Comparative transcriptome analysis and RNA interference reveal CYP6A8 and SNPs related to pyrethroid resistance in Aedes albopictus
Source: PLoS Negl Trop Dis. 2018 Nov 12;12(11):e0006828. doi: 10.1371/journal.pntd.0006828 (PMC6258463; doi:10.1371/journal.pntd.0006828)
Supplement: S4 Table — (DOCX) [file pntd.0006828.s006.docx]

**S6 Table. Knockdown time and mortality rate of *Aedes albopictus* with orally delivered RNAi.**

| Group | Gene targeted by RNAi | n | KDT_50_ (95% CI) | KRR_50_^b^ | 24h corrected mortality rate |
| --- | --- | --- | --- | --- | --- |
| RNAi | *Cyp6a8* | 120 | 16.9 (16.1-17.8) ** | 1.5 (1.4-1.6) ** | 100% ** |
|  | *CCG013931.2* | 100 | 18.5 (17.2,19.8) * | 1.6 (1.5-1.7)* | 100% ** |
|  | *CCG000656.1* | 130 | 16.0 (15.0-17.0) ** | 1.4 (1.3-1.5) ** | 100% ** |
| Control^a^ |  | 120 | 21.2 (19.8-22.6) | 2.1 (2.0-2.2) | 90.5% |

Note: Mortality rate was measured using the standard WHO insecticide susceptibility tube test against 0.05% deltamethrin.

^a^ Control refers to *Ae. albopictus* Lab-DR strain mosquitoes orally treated with a siRNA duplex lacking significant sequence homology to any genes in the *Ae. aegypti* genome.

^b^ KRR_50_ was calculated as the ratio of KDT_50_ of the treatment group to KDT_50_ of the susceptible Foshan mosquito group.

* *P* < 0.05; ** *P* < 0.01 for comparison between RNAi group and control group.
